# Shared and unique common genetic determinants between pediatric and adult celiac disease

**DOI:** 10.1186/s12920-016-0211-8

**Published:** 2016-07-22

**Authors:** Sabyasachi Senapati, Ajit Sood, Vandana Midha, Neena Sood, Suresh Sharma, Lalit Kumar, B. K. Thelma

**Affiliations:** Department of Genetics, University of Delhi South Campus, New Delhi, 110021, India; Department of Gastroenterology, Dayanand Medical College and Hospital, Ludhiana, Punjab India; Department of Medicine, Dayanand Medical College and Hospital, Ludhiana, Punjab India; Department of Pathology, Dayanand Medical College and Hospital, Ludhiana, Punjab India; Department of Nursing, Dayanand Medical College and Hospital, Ludhiana, Punjab India; Present Address: Centre for Human Genetics and Molecular Medicine, Central University of Punjab, Bathinda, Punjab India

**Keywords:** Pediatric celiac disease, Adult celiac disease, Immunochip, *HLA-DQ*, *ANK3*

## Abstract

**Background:**

Based on age of presentation, celiac disease (CD) is categorised as pediatric CD and adult CD. It however remains unclear if these are genetically and/or phenotypically distinct disorders or just different spectrum of the same disease. We therefore explored the common genetic components underlying pediatric and adult CD in a well characterized north Indian cohort.

**Methods:**

A retrospective analysis of children (*n* = 531) and adult (*n* = 871) patients with CD between January 2001 and December 2010 was done. The database included basic demographic characteristics, clinical presentations, associated diseases and complications, if any. The genotype dataset was acquired for children (*n* = 217) and adult CD patients (*n* = 340) and controls (*n* = 736) using Immunochip. Association analysis was performed using logistic regression model to identify susceptibility genetic variants.

**Results:**

The predominant form of CD was classical CD in both pediatric and adult CD groups. There was remarkable similarity between pediatric and adult CD except for quantitative differences between the two groups such as female preponderance, non-classical presentation, co-occurrence of other autoimmune diseases being more common amongst adult CD. Notably, same HLA-DQ2 and –DQ8 haplotypes were established as the major risk factors in both types of CD. In addition, a few suggestively associated (*p* < 5 × 10^−4^) non-HLA markers were identified of which only *ANK3* (rs4948256-A; rs10994257-T) was found to be shared and explain risk for ~45 % of CD patients with HLA allele.

**Discussion:**

Overall phenotypic similarity between pediatric and adult CD groups can be explained by contribution of same HLA risk alleles. Different non-HLA genes/loci with minor risk seem to play crucial role in disease onset and extra intestinal manifestation of CD. None of the non-HLA risk variants reached genome-wide significance, however most of them were shown to have functional implication to disease pathogenesis. Functional relevance of our findings needs to be investigated to address clinical heterogeneity of CD.

**Conclusions:**

This present study is the first comparative study based on common genetic markers to suggest that CD in pediatric age group and in adults are the spectrum of the same disease with novel and shared genetic risk determinants. Follow-up fine mapping studies with larger study cohorts are warranted for further genetic investigation.

**Electronic supplementary material:**

The online version of this article (doi:10.1186/s12920-016-0211-8) contains supplementary material, which is available to authorized users.

## Background

Celiac disease (CD) is a chronic systemic inflammatory condition of autoimmune origin as a result of permanent intolerance to gluten in genetically predisposed individuals [1, 2]. CD was thought initially to be a disease of children and was treated mainly by pediatricians. Over past two decades, it was realized that CD also occurs in adults [3, 4]. Such observations led to the concept of pediatric CD and adult onset CD and it’s sub-clasification based on the age at disease diagnosis [5, 6]. Pediatric CD develops in early years of life with introduction of gluten containing cereals in their diets and presents mostly with classical features of CD such as chronic diarrhoea, abdominal distension, failure to thrive and deficiencies of nutrients. On the other hand, adult CD presents later in life and has both classic manifestations and non-classical manifestation such as fatigue, anaemia, bone disease, idiopathic seizures, cryptogenic liver disease and various autoimmune diseases [7, 8].

The past two decades have witnessed considerable efforts to unravel the pathogenesis of CD including genetic risk determinants [9, 10]. *HLA-DQ2* and *-DQ8* haplotypes have been the most consistently reported genetic markers conferring ~40 % risk for CD development [11, 12]. More recently, an additional 39 loci have been identified by genome-wide association studies (GWAS) and Immunochip based genetic analysis [13]. These additional loci are estimated to confer an additional ~15 % genetic risk to them and explained ~55 % of the CD cases [12]. While all these genetic and clinical studies have brought extremely important information, they however have not delineated phenotypic and genetic characteristics based on the age at the onset leaving significant gaps in our understanding of these two distinct categories. We therefore explored the genetic landscape of a well characterized cohort of pediatric CD and adult CD.

Our observations are suggestive of novel as well as moderately shared genetic determinants underlying these two distinct celiac disease groups.

## Methods

### Study cohort

Database registry system for patients with CD being maintained at the Gastroenterology Department, Dayanand Medical College and Hospital since 1995, was used in this study. A patient registry database containing detailed baseline and follow up clinical and laboratory characteristics of all the patients with confirmed diagnosis of CD.

A retrospective database analysis of all the patients (*n* = 1402) with CD between January 2001 and December 2010 was done. The diagnosis of CD was established as per modified ESPGHAN criteria [14, 15]. Approval for this study was obtained from Institutional Ethics Committee (IEC) of University of Delhi South Campus, New Delhi and Drug Trial Ethics Committee (DTEC), Dayanand Medical College and Hospital, Ludhiana. An informed and written consent was obtained from all the participants. The baseline demographic characteristics such as age at the diagnosis, gender of the participants, and duration of the symptoms prior to diagnosis of CD was retrived from the database. Patients ≥18 years of age at diagnosis were identified as adult CD while patients <18 years were identified as having pediatric CD. Recruitment of pediatric CD patients were guided by “Convention on the Rights of the Child” adopted by the United Nations General Assembly (1989) and Indian Association of Pediatric Policy Statement on Age of Children for Pediatric Care (1999). Patients presenting symptoms such as chronic diarrhea, malabsorption and failure to thrive were classified as Classical CD and those presenting as extra intestinal manifestations such as short stature, fatigue, iron deficiency anaemia, osteoporosis, delayed puberty, infertility, cryptogenic chronic liver disease, idiopathic seizure and dyspepsia not responding to PPI were classified as non-classical CD. Subjects detected to have CD on screening of high-risk group such as family members of index patient, type I diabetes mellitus, cryptogenic liver disease, infertility, thyroiditis were classified as “Screen detected’ [15].

Relevant laboratory data including baseline haemoglobin and anti-tTG antibody levels were recorded. All the patients had undergone gastroduodenoscopic examination and their duodenal biopsies obtained. The grade of villous atrophy was classified as per modified Marsh criteria [16, 17]. All study subjects were from the north part of India and had been residents of the state of Punjab, a State in Northern part of India, for more than three generations.

### Genetic analysis

Of the 1402 CD patients, 557 patients (*n* = 217 pediatric CD and *n* = 340 adult CD) were available for genetic analysis and their DNA was genotyped on the Illumina immunochip genotyping platform [13]. Unrelated healthy blood donors (*n* = 736) from the same locality and community having anti-tTG antibody value within normal range were recruited as controls. They were also genotyped on the Illumina immunochip genotyping platform. The controls were common for both the adults and pediatric patients with CD.

DNA from peripheral whole blood was extracted using conventional phenol-chloroform method at University of Delhi, South Campus, New Delhi, India. Samples were hybridized on the Immunochip platform at the genotyping facility, University Medical Centre Groningen, The Netherlands, as a part of the Celiac Disease Consortium (CDC). Immunochip is a custom made platform with ~186 known immune loci densely covered with 196524 polymorphic variants described before [13, 18]. Total sample set was divided into two groups namely i) Pediatric CD (*n* = 217); and ii) Adult CD (*n* = 340). All the quality control steps, as described elsewhere [13, 18] were performed separately on these two groups. Additionally, all markers with minor allele frequency (MAF) <0.10 were removed. After stringent QC, datasets for Pediatric CD and Adult CD were analysed separately for test of association. Principal component analysis (PCA) was performed using markers in linkage equilibrium (LD < 0.10) to elucidate the underlying genetic stratification present in the study cohort. Principal components 1 to 4 were included as covariates to correct for population stratification and genomic inflation. Since, multiple markers were tested for association in this study (similar to genome-wide association studies), to avoid false positives, Bonferroni correction was applied. Accordingly, significance level was kept at *p* ≤5 × 10^−7^ level and suggestive association at p-values between 5 × 10^−7^ and 5 × 10^−4^. Genetic heterogeneity of odds ratio obtained in test of association (described above) between Pediatric CD and Adult CD were tested using Breslow-Day statistics. Identified variants were further analysed *in silico* for their functional significance by evaluating their expression effects (*cis*-eQTL) on neighbouring genes. Genome-wide whole blood expression data was used, given in Blood eQTL browser [19] to identify *cis*-eQTL effect of the variants. Further, we used GRAIL (Gene Relationships Across Implicated Loci) [20] to understand the possible relationships of the genes identified in our study with already known genes at 39 non-HLA CD loci [13].

### Data availability

Genotyping data for the entire north Indian cohort are available through the European Genome-Phenome Archive (http://www.ebi.ac.uk/ega/) with the accession number EGAS00001000849. The summary statistics can be obtained by contacting the authors directly.

## Results

### Clinical characteristics

Of 1402 patients with CD in our database, 531 (37.8 %) were pediatric CD and 871 (62.2 %) were adult CD. The mean age at the diagnosis in pediatric CD and adult CD was 10.08 ± 4.6 years (females 45.4 %) and 35.34 ± 11.83 years (females 60.3 %). The duration of symptoms before the diagnosis of CD was comparable in adult CD in comparison to that in pediatric CD (3.31 years versus 2.2 years; *p* = NS). While the predominant mode of presentation in both pediatric and adult CD was classical CD; the proportion of patients with classical CD was higher in pediatric CD in comparison of that in adult CD (75.5 % vs 69.6 %; *p* = 0.009). The non-classical presentation was statistically higher in adult CD in comparison to that in pediatric CD (20.3 % vs 25.2 %; *p* = 0.02).

Iron deficiency anemia and hypothyroidism were more commonly reported in adult CD (Table 1). Complications such as celiac crisis, refractory sprue and associated malignancy were seen in the adult group. The various malignancies reported included oesophageal, colon, stomach and small bowel sarcoma (one each). Dermatitis herpetiformis was seen in 3 adult CD cases while none was observed in pediatric CD. Associated autoimmune disorders, investigations, titre of anti-tTG antibody in pediatric and adult CD are summarized in Table 1.

**Table 1:** Demographic and clinical characteristics of pediatric and adult celiac disease

Variables	Pediatric CD patients (*n* = 531)	Adults CD patients (*n* = 871)	*P* value
Mean ± SD age (in years)	10.08 ± 4.60	35.34 ± 11.83	NA
Gender			
Male Female	290 (54.61) 241 (45.40)	345 (39.60) 526 (60.40)	**1 × 10** ^**−3**^ **1 × 10** ^**−3**^
Presentation			
• Classical • Non-classical • Screening	401 (75.50) 108 (20.30) 22 (4.20)	606 (69.60) 220 (25.20) 45 (5.20)	**0.01** **0.02** 0.13
Associated diseases			
Iron deficiency anaemia Short stature Osteoporosis Raised transaminases Autoimmune hepatitis Infertility Hypothyroidism Type 1 diabetes Sjogren’s syndrome Dermatitis herpetiformis Idiopathic seizure Down’s syndrome Inflammatory bowel disease Alopecia areata Rheumatoid arthritis Vitiligo Primary biliary cirrhosis	72(13.20) 17 (3.20) 0 (0.00) 28(5.27) 03(0.56) NA(0.00) 02(0.38) 05(0.94) 0(0.00) 0(0.00) 01(0.19) 01(0.19) 01(0.19) 01(0.19) 0(0.00) 0(0.00) 0(0.00)	167 (19.20) 37(4.25) 06(0.69) 58(6.60) 06(0.69) 03(0.34) 17(1.95) 03(0.34) 05(0.57) 03(0.34) 06(0.69) 02(0.23) 03(0.34) 0(0.00) 3(0.34) 3(0.34) 3(0.34)	**0.01** 0.17 **0.05** 0.16 0.59 0.12 **0.04** 0.12 0.10 0.12 0.13 0.82 0.31 0.13 0.12 0.12 0.12
Complications			
Celiac crisis Refractory CD Malignancy	---- ---- ----	04 (0.5) 02 (0.3) 04 (0.5)	**1 × 10** ^**−3**^ **1 × 10** ^**−3**^ **1 × 10** ^**−3**^
Mean titre of anti-tTG antibody (U/L)	130.00	118.60	**1 × 10** ^**−3**^
Grade of villous abnormalities (Modified Marsh classification)			
I/II IIIa IIIb IIIc	26 (4.63) 44 (8.28) 115 (21.65) 346 (65.16)	92 (10.56) 139 (15.95) 170 (19.51) 470 (53.96)	**7 × 10** ^**−3**^ **6 × 10** ^**−3**^ 0.17 **6 × 10** ^**−3**^

*NA* not applicable, The values presented in parenthesis is a percentages

Bolds (*P* values) are the significant associations (*p* ≤ 0.05)

### Genetic analysis

After stringent quality control of the immunochip based genotype data, a total of 27 pediatric CD, 33 adult CD and 6 controls were removed. Following removal of poorly clustered markers due to poor hybridization, 172,242 markers were considered for further analysis. Of them, 88,776 and 88,793 markers in Pediatric CD and Adult CD groups passed the QC steps. The genomic control inflation factor of association test statistics (λ_GC_) was observed to be very limited in the two groups analysed in the study (λ_GC_ = 1.11 for pediatric CD; and λ_GC_ = 1.01 for adult CD) (Additional file 1: Figure S1a and b). Notably, Pediatric CD and Adult CD had 90 % and 95 % power, respectively to detect association of a variant having minor allele frequency (MAF) >0.20 and odds ratio (OR) >1.50.

### Test of association

*HLA* locus emerged as the most significantly associated marker both in pediatric and adult CD patients (Tables 2 and 3; Additional file 1: Figure S2a and b). The strongest signal was at rs2854275, G > T, which is localized in the last intron of *HLA-DQB1*and which is in very strong LD (*r2* = 0.95, D’ = 1) with European top HLA marker rs2187668 from *HLA-DQA1,* was common in both pediatric CD (*p* = 4.28 × 10^−29^) and adult CD (*p* = 5.82 × 10^−35^). A frequency of 14.9 % at rs2854275-T allele in healthy individuals versus 49.7 % in adult CD and 50 % in pediatric CD patients was noteworthy. Furthermore, while conditioned on rs2854275, we did not observe any other genome-wide significant HLA marker in either pediatric or adult CD groups. Association status of five known SNPs (rs2395182, rs7775228, rs4713586, rs2187668 and rs7454108) tagging HLA-DQ haplotypes [21] were checked in pediatric CD and adult CD. Comparable allelic association status for risk alleles (Table 4) and distribution of different-HLA-DQ haplotypes (Table 5) were observed between these two groups. We observed that similar proportion of CD patients in both these groups (87.30 % in Pediatric CD and 86.97 % in Adult CD) had at least one of the two risk haplotypes HLA-DQ2.5 or -DQ8, which was also comparable that reported in the European populations [22–24].

**Table 2:** Top association signals identified in Pediatric CD and their association status in Adult CD among north Indians patients

Pediatric CD					Adult CD			
SNPs	(RA)	RAF	OR	*P*	OR	*P*	Position	Ref Seq genes
rs2854275	T	0.15	6.22[4.52–8.57]	4.28 × 10^−29^	6.11[4.58–8.15]	5.82 × 10^−35^	intronic	*HLA-DQB1*
rs4948256	A	0.22	1.90[1.46–2.48]	2.06 × 10^−6^	1.50[1.18–1.90]	1 × 10^−3^	intronic	*ANK3*
rs1322423	G	0.87	2.49[1.58–3.94]	8.75 × 10^−5^	1.05[0.77–1.41]	0.77	694 kb 3’	*TSG1*
rs4925813	G	0.51	1.64[1.28–2.10]	1.03 × 10^−4^	1.28[1.04–1.58]	0.02	17 kb 5’	*ARHGAP39*
rs956506	T	0.23	1.68[1.28–2.20]	1.79 × 10^−4^	1.20[0.94–1.54]	0.14	intronic	*MACROD2*
rs2981579	C	0.57	1.60[1.25–2.08]	2.54 × 10^−4^	1.05[0.85–1.29]	0.64	intronic	*FGFR2*
rs4806741	G	0.12	1.84[1.32–2.56]	3.17 × 10^−4^	1.41[1.03–1.94]	0.03	6.1 kb 5'	*LILRA3*
rs854680	T	0.29	1.58[1.23–2.04]	3.60 × 10^−4^	1.09[0.87–1.37]	0.45	527 bp 5'	*CCL16*
rs4692386	T	0.50	1.56[1.22–1.99]	3.64 × 10^−4^	0.93[0.75–1.15]	0.50	189 kb 5'	*RBPJ*
rs9300549	G	0.61	1.64[1.25–2.14]	3.64 × 10^−4^	0.85[0.68–1.06]	0.16	145 kb 5'	*GRP183*
rs4857943	G	0.29	1.57[1.22–2.01]	3.83 × 10^−4^	1.20[0.95–1.50]	0.12	178 kb 3'	*SGOL1*
rs10503653	C	0.27	1.60[1.23–2.07]	3.87 × 10^−4^	1.27[1.01–1.60]	0.05	intronic	*CSGALNACT1*
rs2061737	G	0.51	1.58[1.23–2.03]	3.89 × 10^−4^	0.91[0.74–1.23]	0.38	3q26.1	*N/A*

*RA* risk allele, *RAF* risk allele frequency in controls

**Table 3:** Top association signals identified in Adult CD group and their association status in Pediatric CD among north Indians

Adult CD					Pediatric CD			
SNPs	(RA)	RAF	OR	*P*	OR	*P*	Position	Ref Seq genes
rs2854275	T	0.15	6.11[4.58–8.15]	5.82 × 10^−35^	6.22[4.52–8.57]	4.28 × 10^−29^	intronic	*HLA-DQB1*
rs2788057	T	0.44	1.56[1.27–1.92]	2.29 × 10^−5^	1.20[0.95–1.53]	0.13	intronic	*NMNAT2*
rs11119390	C	0.74	1.72[1.33–2.23]	3.83 × 10^−5^	1.14[0.86–1.49]	0.36	intronic	*MAPKAPK2*
rs9359383	G	0.14	1.82[1.37–2.42]	4.05 × 10^−5^	1.39[0.99–1.94]	0.06	12 kb 5’	*LCA5*
rs11074015	T	0.45	1.55[1.25–1.92]	7.86 × 10^−5^	1.01[0.80–1.28]	0.93	126.6 kb 5'	*SLCO3A1*
rs422999	C	0.19	1.65[1.29–2.12]	8.21 × 10^−5^	1.34[1.00–1.78]	0.05	intronic	*VAV2*
rs1875897	G	0.57	1.56[1.25–1.95]	1.06 × 10^−4^	1.17[0.91–1.50]	0.22	intronic	*CSMD1*
rs2332094	A	0.12	1.79[1.33–2.41]	1.28 × 10^−4^	1.27[0.88–1.82]	0.20	8.6 kb 5'	*KIAA0247*
rs11220082	C	0.32	1.53[1.23–1.90]	1.38 × 10^−4^	1.04[0.81–1.35]	0.76	intronic	*FEZ1*
rs1999594	C	0.33	1.52[1.22–1.88]	1.68 × 10^−4^	1.11[0.86–1.43]	0.40	20.4 kb 3'	*KIAA2013*
rs1870162	C	0.08	1.85[1.33–2.55]	2.17 × 10^−4^	N/A	N/A	intronic	*GRID1*
rs35164067	A	0.20	1.60[1.25–2.06]	2.28 × 10^−4^	1.23[0.92–1.65]	0.17	6.1 kb 5'	*PDE4A*
rs17463487	T	0.65	1.53[1.22–1.92]	2.23 × 10^−4^	1.10[0.85–1.41]	0.47	131.6 kb 3'	*ZNF326*
rs6720771	G	0.86	1.73[1.29–2.32]	2.42 × 10^−4^	1.16[0.85–1.59]	0.34	intronic	*GALNT13*
rs10141085	G	0.18	1.62[1.25–2.10]	2.44 × 10^−4^	0.95[0.69–1.31]	0.77	intronic	*KIAA0391*
rs4897233	G	0.40	1.49[1.20–1.84]	2.50 × 10^−4^	1.24[0.97–1.58]	0.09	intronic	*THEMIS*
rs1399357	C	0.51	1.49[1.20–1.85]	2.56 × 10^−4^	1.29[1.01–1.64]	0.04	363 kb 3'	*FAM181B*
rs963872	C	0.42	1.48[1.19–1.83]	3.38 × 10^−4^	1.32[1.03–1.69]	0.03	5p15.33	N/A
rs10994257	T	0.26	1.51[1.20–1.90]	4.00 × 10^−4^	1.62[1.25–2.09]	2.48 × 10^−4^	intronic	*ANK3*
rs12730072	T	0.09	1.76[1.29–2.41]	4.10 × 10^−4^	1.67[1.14–2.42]	8 × 10^−3^	229 bp 5'	*ITLN2*

*RA* risk allele, *RAF* risk allele frequency in controls

**Table 4:** Comparative association status of known tag SNPs (Monsur et al., [21], Romanos et al., [11] and [12]) for DQ haplotypes

HLA alleles	Risk allele (RA)	Risk allele frequency			*P*-value	OR	CD groups
		Indian	African	European			
HLA-DQ2.2 (rs2395182)	T	0.83	0.73	0.76	1.06 × 10^−5^	2.72 [1.74–4.25]	Pediatric
					6.26 × 10^−5^	1.99 [1.42–2.79]	Adult
HLA-DQ2.2 (rs7775228)	T	0.73	0.83	0.85	1.14 × 10^−4^	1.89 [1.36–2.63]	Pediatric
					5.08 × 10^−7^	2.12 [1.58–2.84]	Adult
HLA-DQ2.5 (rs2187668)	A	0.16	0.06	0.10	3.46 × 10^−28^	6.03 [4.38–8.30]	Pediatric
					2.45 × 10^−34^	5.99 [4.49–7.98]	Adult
HLA-DQ8 (rs7454108)	T	0.94	0.97	0.89	0.08	1.72 [0.93–3.18]	Pediatric
					0.20	1.37 [0.84–2.22]	Adult
HLA-DQ4 (rs4713586)	T	0.98	0.92	0.94	0.03	9.05 [1.18–69.50]	Pediatric
					0.03	3.71 [1.18–11.78]	Adult

Genotype data for SNP (rs4639334) tagging -DQ7 haplotype was not present in the dataset and therefore was not available for the study

**Table 5:** Comparative distribution of HLA-DQ haplotypes in pediatric CD and adult CD groups

HLA genotypes	PediatricCD	AdultCD
DQ2.5/DQ8	9	15
DQ2.5/DQ2.2	37	50
DQ2.5/DQX	114	192
DQ2.2/DQ8	1	1
DQ2.2/DQX	15	25
DQ8/DQX	4	9
DQX/DQX	9	14

Prediction of DQ genotypes were done based on known tag SNPs originally identified in Europeans. One individual from each group was dropped from the study as genotype information for all tag SNPs were not present in the final dataset for these two individuals

On the other hand, 12 distinct non-HLA risk variants were observed to be suggestively associated (p <5 × 10^−4^) with pediatric CD and 19 variants with adult CD with only *ANK3* gene being common to both groups (Tables 2 and 3). Though most of these are suggestive associations for CD, a few of them are known risk loci for other immune disorders (Additional file 2: Table S1). Except for *ANK3* [ankyrin 3], *ARHGAP39* [Rho GTPase activating protein 39]*, LILRA3* [leukocyte immunoglobulin-like receptor, superfamily A (without TM domain), member 3] and *CSGALNACT1* (chondroitin sulfate N-acetylgalactosaminyltransferase 1] other Pediatric CD specific loci were not replicated (*p* ≤ 0.05) in Adult CD. Similarly, only four Adult CD specific loci, namely *VAV2* [vav 2 guanine nucleotide exchange factor], *FAM181B* [family with sequence similarity 181, member B], non-genic region at 5p15.33, *ANK3* [ankyrin 3] and *ITLN2* [intelectin 2] were replicated in pediatric CD. Further ~58 % Pediatric CD specific variants were heterogeneous (p_BD < 0.05) compared to ~26 % among adult CD specific variants (Additional file 2: Table S2). cis-eQTL analysis revealed five variants in the Pediatric CD group and six in the Adult CD group to be functionally relevant (Additional file 2: Table S3). GRAIL analysis revealed three loci, two from Pediatric CD and one from Adult CD to be significantly connected with the known 39 non-HLA CD loci (Additional file 2: Table S4).

## Discussion

Celiac disease is one of the complex disorder where the major non-genetic factor namely gluten has been very well established. Further, two distinct groups of affected individuals based on age at onset of the disease have also been noted. The major limitation to date has however been the lack of information in the similarities and differences, if any, in the genetic susceptibility of the two groups. Analysis of clinical characteristics in this study cohort has demonstrated that while classic CD is the predominant manifestation in both pediatric and adult CD, non-classical manifestations are also commonly present in both the groups, iron deficiency anaemia being the most frequent (Table 1). An association has been reported between other autoimmune disorders and adult CD as approximately 30 % of adult patients with CD had one or more associated autoimmune disorders in comparison to approximately 3 % in the general population [25–27]. This may happen due to late diagnosis of CD and longer duration of exposure to gluten in adults [25]. On the other hand, early institution of gluten-free diet in children patients may act as a protective factor against development of another autoimmune disease in pediatric CD [28].

It is noteworthy that already reported *HLA-DQ* haplotypes emerged as the major genetic risk factor in both pediatric and adult CD groups (Table 4) and confers equal risk to both of these groups (Table 5). In addition *ANK3*, an integral membrane protein controlling cytoskeleton, cell motility, proliferation and intestinal contraction, was the only other novel non-HLA marker which was associated with both pediatric and adult CD groups (Tables 2 and 3). Along with top HLA marker (rs2854275-T), risk variants from *ANK3* (rs4948256-A among pediatric and rs10994257-T among adults) explains ~45 % of the CD cases. This finding reiterates our previous observation in a trans-ethnic study, where *ANK3* was identified in the same combined CD cohort [18]. It is worthwhile to mention that *ANK3* has been shown to be associated with bipolar disorder and schizophrenia [29–32]. Interestingly, a co-occurrence of bipolar disorder and schizophrenia has been observed in patients with CD [33–35]. Furthermore, approximately 10 % of patients with CD have some form of neurological disorders such as seizures, cognitive impairment, migraine or other psychiatric illness [33, 34] However, role if any, of *ANK3* in occurrence of neurological and psychological disorder in patients with CD warrant further investigations. Contrary to two (*HLA-DQ* and *ANK3*) shared genes, most of the other associated genes in both pediatric and adult CD are notably different from each other. Though these genes seem functionally related and are present in similar pathways including cell signalling, cell cycle regulation, inflammatory responses and cellular metabolism (Additional file 2: Table S1). The degree of risk for associated allelic variants with pediatric CD is significantly different (higher degree of odds ratio heterogeneity) from the allelic variants in the adult CD groups (Additional file 2: Table S2). This may signify that these genes with minor risk contributions are specific to each of these two groups and might work in a network with known susceptible genes for the onset of different clinical phenotypes. In the absence of literature on the detailed function of these novel genes, their exact role in age specific CD pathogenesis remains to be understood.

Of 39 known non-HLA CD loci [13], *THEMIS*, a gene involved in T-cell development was observed to be associated with only adult CD group. Cis-eQTL evaluation of the suggestive risk variants that we observed in the study, indicated highly significant functional relevance of 11 (35.5 %) risk variants which were seen to alter the level of gene expression at these loci (Additional file 2: Table S3). Further, pathway and genetic interaction analysis identified *LILRA3, CCL16* and *VAV2* which are known to be involved in inflammatory process and are functionally related to the known 39 non-HLA CD loci (Additional file 2: Table S4). This novel comparative study identified suggestive loci in each of these groups but needs follow-up replication. *In silico* evidences of their effects on gene expression and involvement in integrated pathways in disease pathogenesis support their probable contribution. Therefore, additional investigations across ethnic groups and also functional validation are warranted.

## Conclusions

Pediatric and adult CD seem to be a continuum of the disease entity with same HLA-DQ haplotypes and *ANK3* being major genetic risk determinants. However, different sets of minor risk conferring genetic loci with promising functional involvement but which are present in a common network with other genes may determine the quantitative disease heterogeneity between them. These, preliminary findings warranted replication and fine mapping to confirm risk alleles.

## Abbreviations

CD, Celiac disease; CDC, Celiac disease consortium; eQTL, expression quantitative trait loci; ESPGHAN, The European Society for Paediatric Gastroenterology Hepatology and Nutrition; GRAIL, Gene Relationship Across Implicated Loci; GWAS, genome-wide association study; LD, linkage disequilibrium; MAF, minor allele frequency; OR, odds ratio; PCA, principal component analysis; PPI, proton-pump inhibitors; tTG, tissue transglutaminase

## Additional files

Additional file 1:
**Figure S1.** QQ plots showing level of genomic inflation in (a) Paediatric CD and (b) Adult CD groups in north Indian population. Inflation was measured using 3016 independent, neutral-reported variants present on the array (derived from reading and math skills GWAS therefore unlikely to be confounded by the immune signal). **FigureS2.** Manhattan plots showing association signals in (a) Paediatric CD and (b) Adult CD in north Indian population. (DOCX 254 kb) Additional file 2:
**Table S1.** Functional profiles of the top non-HLA association signals identified in Paediatric CD and Adult CD among north Indians. **Table S2.** Test of heterogeneity (Breslow-Day test) for associated SNPs in PaediatricCD and AdultCD groups. **Table S3.** *cis*-eQTL evaluation of associated SNPs. **Table S4.** GRAIL analysis revealed seven genes with significant (*p* <0.05) interaction with 39 known non-HLA coeliac disease loci. These seven genes are from four loci identified in this study. (DOCX 26 kb)
