# Supplementary material for: Shared and unique common genetic determinants between pediatric and adult celiac disease
Source: BMC Med Genomics. 2016 Jul 22;9:44. doi: 10.1186/s12920-016-0211-8 (PMC4957920; doi:10.1186/s12920-016-0211-8)

**Supplementary Figure1:** QQ plots showing level of genomic inflation in (a) Paediatric CD and (b) Adult CD groups in north Indian population. Inflation was measured using 3016 independent, neutral-reported variants present on the array (derived from reading and math skills GWAS therefore unlikely to be confounded by the immune signal).

**
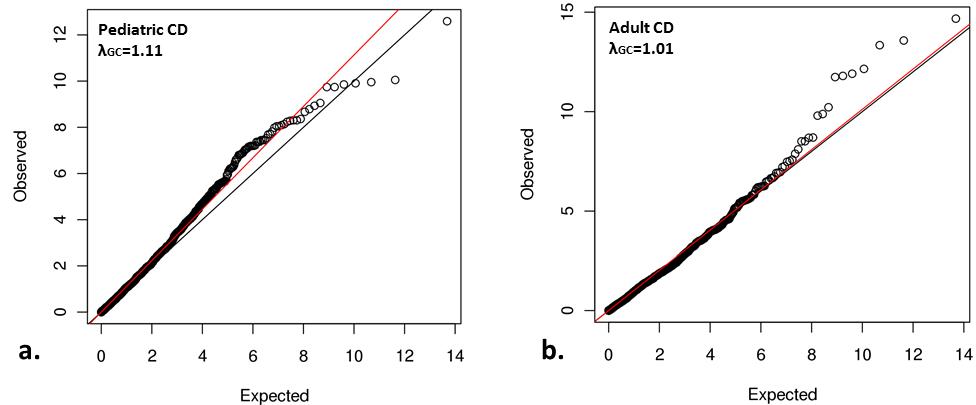
**

**Supplementary Figure2:** Manhattan plots showing association signals in (a) Paediatric CD and (b) Adult CD in north Indian population


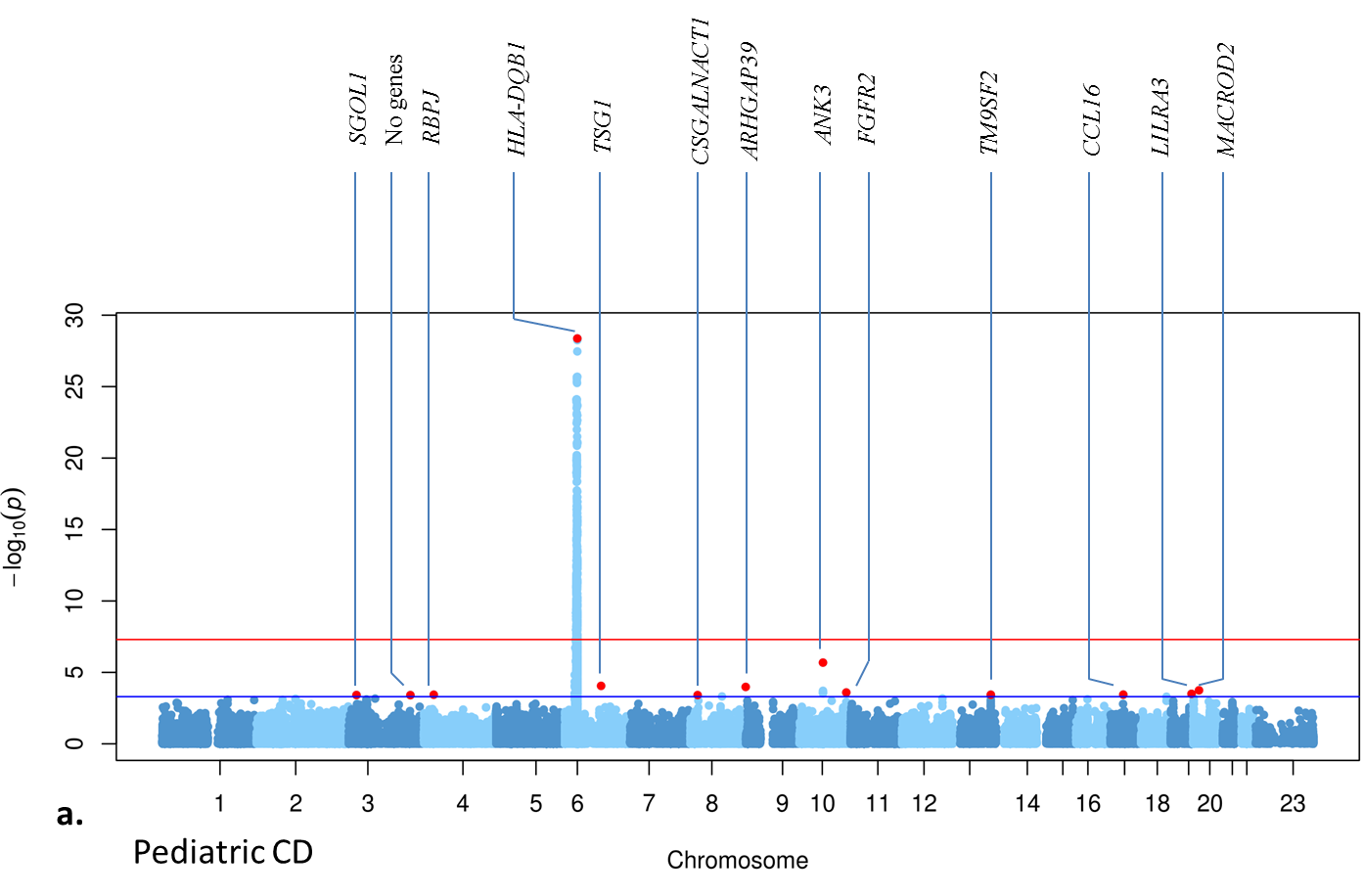


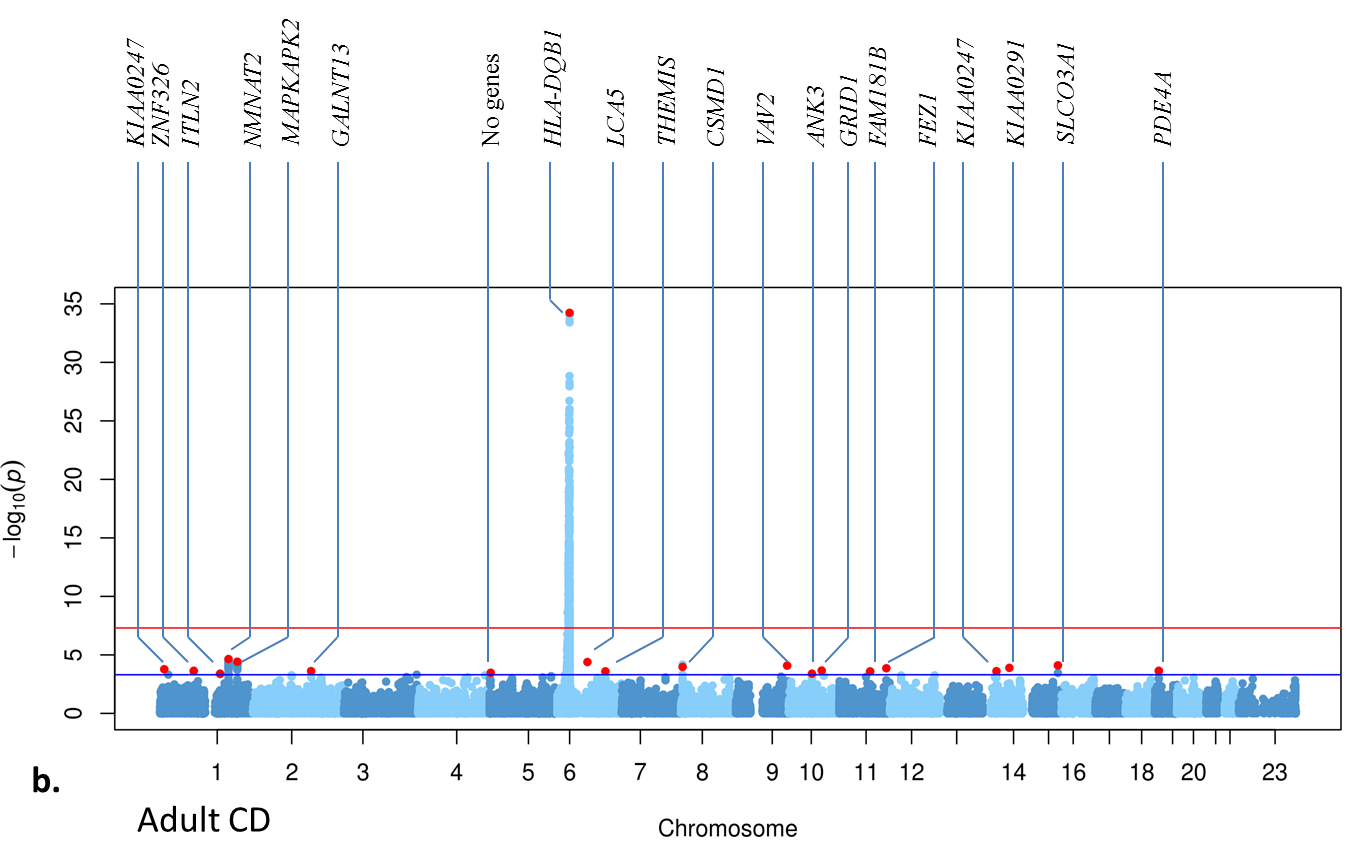

Supplement: Additional file 1: — Figure S1. QQ plots showing level of genomic inflation in (a) Paediatric CD and (b) Adult CD groups in north Indian population. Inflation was measured using 3016 independent, neutral-reported variants present on the array (derived from reading and math skills GWAS therefore unlikely to be confounded by the immune signal). FigureS2. Manhattan plots showing association signals in (a) Paediatric CD and (b) Adult CD in north Indian population. (DOCX 254 kb) [file 12920_2016_211_MOESM1_ESM.docx]
