# Supplementary material for: Shared and unique common genetic determinants between pediatric and adult celiac disease
Source: BMC Med Genomics. 2016 Jul 22;9:44. doi: 10.1186/s12920-016-0211-8 (PMC4957920; doi:10.1186/s12920-016-0211-8)
Supplement: Additional file 2: — Table S1. Functional profiles of the top non-HLA association signals identified in Paediatric CD and Adult CD among north Indians. Table S2. Test of heterogeneity (Breslow-Day test) for associated SNPs in PaediatricCD and AdultCD groups. Table S3. cis-eQTL evaluation of associated SNPs. Table S4. GRAIL analysis revealed seven genes with significant (p <0.05) interaction with 39 known non-HLA coeliac disease loci. These seven genes are from four loci identified in this study. (DOCX 26 kb) [file 12920_2016_211_MOESM2_ESM.docx]

**Supplementary Table 1:** Functional profiles of the top non-HLA association signals identified in PaediatricCD and AdultCD among north Indians

| **Genes** | | **Known associations** | | **Functions** |
| --- | --- | --- | --- | --- |
| *ANK3* | | Schizophrenia, Bipolar disorder | | Localized at plasma membrane and found primarily at the axonal initial segment and nodes of Ranvier of neurons in the central and peripheral nervous system including intestinal nervous system. It links integral membrane proteins to cytoskeleton and helps in cell motility, proliferation and specialized membrane domain. Recently, expression reported in neuromuscular junctions and helps in intestinal muscular contractions. |
| *TSG1* | | Prostate cancer | | Tumor suppressor gene. Multiple splice variants present and its expression is known to be induced by androgen ablation. |
| *ARHGAP39* | | N/A | | A Rho GTPase activating protein involved in many cellular signaling processes |
| *MACROD2* | | Nonalcoholic fatty liver disease, Autistic disorder, Obesity, Carcinoid tumor | | Present in cytoplasm and nucleus. It has hydrolase activity on glycosyl bonds and deacetylase activity |
| *FGFR2* | | Breast neoplasm | | Member of fibroblast growth factor family and have multiple transcript variants. Upon stimulation influence downstream signaling pathway leading to mitogenesis and differentiation |
| *LILRA3* | | Blood lipid | | Cell surface receptor but lacks transmembrane region and acts as a soluble antagonist to *LILRA1* and *LILRA2*.Can act as anti-inflammatory protein. |
| *CCL16* | | N/A | | This cytokine acts as potent myelosuppressor and suppresses proliferation of myeloid progenitor cells. IL-10 regulates the expression of this gene |
| *RBPJ* | | Rheumatoid arthritis | | It is a transcriptional regulator. Regulates Notch protein activation by chromatin remodelling |
| *GRP183* | | Inflammatory bowel disease | | A G-protein coupled receptor, expressed in B lymphocytes and lymphoid tissues but not in T-lymphocytes. |
| *SGOL1* | | Bipolar disease | | Primarily localized in chromosomes, this protein influence chromosomal instability and cell cycle mechanism |
| *CSGALNACT1* | | N/A | | Ubiquitously expressed integral membrane protein |
| *NMNAT2* | N/A | | Ubiquitously expressed cytoplasmic protein catalyze an essential step in NADP biosynthesis pathway | |
| *MAPKAPK2* | N/A | | A serine/threonine kinase involved in many cellular process including stress and inflammatory responses, nuclear export, gene expression regulation and cell proliferation | |
| *LCA5* | Antiphospholipid syndrome | | This protein thought to be involved in centrosomal or ciliary function | |
| *SLCO3A1* | Childhood acute lymphoblastic leukemia, Attention deficit hyperactivity disorder | | A membrane transport protein helps in auxiliary transport | |
| *VAV2* | Multiple sclerosis | | Guanine nucleotide exchange factor helps is cellular communication and signal transduction | |
| *CSMD1* | Cognition, Multiple sclerosis, Emphysema, Schizophrenia, Bipolar disorder | | Integral membrane protein functions in cell communication and signal transduction | |
| *KIAA0247* | N/A | | Integral membrane protein known to be a cell cycle modulator | |
| *FEZ1* | Schizophrenia | | This protein operates as a kinesin adaptor | |
| *KIAA2013* | N/A | | Not well known | |
| *GRID1* | Cardiac health | | Integral membrane protein, a glutamate receptor channel. These channels mediate most of the fast excitatory synaptic transmission in the CNS and play key roles in synaptic plasticity | |
| *PDE4A* | N/A | | A phosphodiesterase which hydrolyzescAMP and thus regulates a wide range of cellular processes | |
| *ZNF326* | N/A | | A DNA binding protein, regulates nucleobase, nucleoside, nucleotide and nucleic acid metabolism | |
| *GALNT13* | Longevity, Sudden cardiac arrest | | A galactosyltransferase protein which initiate O-linked glycosylation of mucins | |
| *KIAA0391* | Psoriasis | | Not well known | |
| *THEMIS* | Multiple sclerosis, Coeliac disease | | Encoded protein plays a regulatory role in both positive and negative T-cell selection during late thymocyte development. This protein functions through T-cell antigen receptor signalling | |
| *FAM181B* | N/A | | Not well known | |
| *ITLN2* | N/A | | Not well known | |

**Supplementary Table 2:** Test of heterogeneity (Breslow-Day test) for associated SNPs in PaediatricCD and AdultCD groups

| **PaediatricCD** | | | | |
| --- | --- | --- | --- | --- |
| **Chr.** | **SNP** | **bp (hg19)** | **p_BD** | **Genes** |
| 6 | rs2854275 | 32628428 | 0.789 | *HLA-DQB1* |
| 10 | rs4948256 | 61956912 | 0.110 | *ANK3* |
| 6 | rs1322423 | 95179912 | 8.7x10^-4^ | *TSG1* |
| 8 | rs4925813 | 145856021 | 0.299 | *ARHGAP39* |
| 20 | rs956506 | 15608581 | 0.061 | *MACROD2* |
| 10 | rs2981579 | 123337335 | 0.022 | *FGFR2* |
| 19 | rs4806741 | 54810370 | 0.173 | *LILRA3* |
| 17 | rs854680 | 34309051 | 0.038 | *CCL16* |
| 4 | rs4692386 | 26132361 | 2.0x10^-3^ | *RBPJ* |
| 13 | rs9300549 | 100103734 | 4.1x10^-4^ | *GRP183* |
| 3 | rs4857943 | 20405255 | 0.026 | *SGOL1* |
| 8 | rs10503653 | 19330935 | 0.096 | *CSGALNACT1* |
| 3 | rs2061737 | 162238239 | 2.2x10^-3^ | *N/A* |
| **AdultCD** | | | | |
| **Chr** | **SNP** | **bp (hg19)** | **p_BD** | **Genes** |
| 1 | rs2788057 | 183345707 | 0.088 | *NMNAT2* |
| 1 | rs10863805 | 206904464 | 0.025 | *MAPKAPK2* |
| 6 | rs9359383 | 80258802 | 0.257 | *LCA5* |
| 15 | rs11074015 | 92270337 | 0.020 | *SLCO3A1* |
| 9 | rs422999 | 136844754 | 0.338 | *VAV2* |
| 8 | rs1875897 | 4188891 | 0.044 | *CSMD1* |
| 14 | rs2332094 | 70069672 | 0.128 | *KIAA0247* |
| 11 | rs11220082 | 125323965 | 0.012 | *FEZ1* |
| 1 | rs1999594 | 11959216 | 0.079 | *KIAA2013* |
| 10 | rs1870162 | 87991098 | N/A | *GRID1* |
| 19 | rs35164067 | 10525181 | 0.179 | *PDE4A* |
| 1 | rs17463487 | 90625731 | 0.079 | *ZNF326* |
| 2 | rs6720771 | 154753536 | 0.093 | *GALNT13* |
| 14 | rs10141085 | 35594442 | 0.013 | *KIAA0391* |
| 6 | rs4897233 | 128156888 | 0.480 | *THEMIS* |
| 11 | rs1399357 | 82080010 | 0.417 | *FAM181B* |
| 5 | rs963872 | 2857542 | 0.478 | N/A |
| 10 | rs10994257 | 61982048 | 0.433 | *ANK3* |
| 1 | rs12730072 | 160924819 | 0.730 | *ITLN2* |

**Supplementary Table 3:** *cis*-eQTL evaluation of associated SNPs

| **SNPs** | **Minor allele** | **P-value** | **Z-score** | **Gene name** | **FDR** | **Known associations** |
| --- | --- | --- | --- | --- | --- | --- |
| **PaediatricCD** | | | | | | |
| rs4925813 | A | 4.21x10^-69^ | -17.57 | *LRRC24, LRRC14* | 0.00 | -- |
| rs4806741 | G | 2.99x10^-41^ | -13.45 | *LILRA5* | 0.00 | -- |
| rs9300549 | G | 9.90x10^-4^ | 3.29 | *GPR18, PHGDHL1* | 0.24 | -- |
| rs4857943 | G | 6.75x10^-16^ | -8.08 | *KAT2B* | 0.00 | SEZ, BD |
| rs10503653 | G | 7.81x10^-6^ | -4.47 | *CSGALNACT1* | 0.00 | Neuropathies |
| **AdultCD** | | | | | | |
| rs2788057 | A | 3.94x10^-65^ | 17.04 | *SMG7* | 0.00 | -- |
| rs2332094 | A | 1.14x10^-13^ | -7.42 | *KIAA0247* | 0.00 | colorectal cancer |
| rs11220082 | C | 2.67x10^-7^ | -5.15 | *FEZ1* | 0.00 | SCZ |
| rs1999594 | G | 1.55x10^-74^ | 18.27 | *PLOD1* | 0.00 | -- |
| rs10141085 | G | 1.73x10^-14^ | -7.67 | *KIAA0391* | 0.00 | PS |
| rs12730072 | T | 4.40x10^-51^ | 15.03 | *USF1* | 0.00 | IBD |

**Supplementary Table 4:** GRAIL analysis revealed seven genes with significant (p<0.05) interaction with 39 known non-HLA coeliac disease loci. These seven genes are from four loci identified in this study

| **SNPs** | **Chr.** | **Locus range (start-end)** | **All genes present at the locus** | **GRAIL p-value** |
| --- | --- | --- | --- | --- |
| rs4806741 | 19 | 54754688-54844688 | *LILRA6, LILRA5, LILRA3, LILRB5, LILRB2* | 9.7x10^-4^ (*LILRB2*) |
| rs854680 | 17 | 34308387-34352887 | *CCL23, CCL16, CCL15, CCL14* | 4.36x10^-5^ (*CCL16*);  1.5x10^-3^ (*CCL23*);  2.2x10^-3^ (*CCL15*);  0.03 (*CCL14*) |
| rs422999 | 9 | 136835446-136854946 | *VAV2* | 5.0x10^-3^ (*VAV2*) |
